# Dichotomous roles for the orphan nuclear receptor NURR1 in breast cancer

**DOI:** 10.1186/1471-2407-13-139

**Published:** 2013-03-21

**Authors:** Shawn Llopis, Brittany Singleton, Tamika Duplessis, Latonya Carrier, Brian Rowan, Christopher Williams

**Affiliations:** 1 Division of Basic Pharmaceutical Sciences, College of Pharmacy, Xavier University of Louisiana, 1 Drexel Dr, New Orleans, LA, 70125, USA; 2 Department of Structural and Cellular Biology, Tulane University School of Medicine, 1483 Tulane Av, New Orleans, LA, 70118, USA

**Keywords:** Breast cancer, NURR1, NR4A2, Orphan receptor

## Abstract

**Background:**

NR4A orphan nuclear receptors are involved in multiple biological processes which are important in tumorigenesis such as cell proliferation, apoptosis, differentiation, and glucose utilization. The significance of NR4A family member NURR1 (NR4A2) in breast cancer etiology has not been elucidated. The purpose of this study was to ascertain the impact of NURR1 expression on breast transformation, tumor growth, and breast cancer patient survival.

**Methods:**

We determined the expression of NURR1 in normal breast versus breast carcinoma in tissue microarrays (immunohistochemistry), tissue lysates (immunoblot), and at the mRNA level (publically available breast microarrays). In addition NURR1 expression was compared among breast cancer patients in cohorts based on p53 expression, estrogen receptor α expression, tumor grade, and lymph node metastases. Kaplan-Meier survival plots were used to determine the correlation between NURR1 expression and relapse free survival (RFS). Using shRNA-mediated silencing, we determined the effect of NURR1 expression on tumor growth in mouse xenografts.

**Results:**

Results from breast cancer tissue arrays demonstrate a higher NURR1 expression in the normal breast epithelium compared to breast carcinoma cells (p ≤ 0.05). Among cases of breast cancer, NURR1 expression in the primary tumors was inversely correlated with lymph node metastases (p ≤ 0.05) and p53 expression (p ≤ 0.05). Clinical stage and histological grade were not associated with variation in NURR1 expression. In gene microarrays, 4 of 5 datasets showed stronger mean expression of NURR1 in normal breast as compared to transformed breast. Additionally, NURR1 expression was strongly correlated with increase relapse free survival (HR = 0.7) in a cohort of all breast cancer patients, but showed no significant difference in survival when compared among patients whom have not been treated systemically (HR = 0.91). Paradoxically, NURR1 silenced breast xenografts showed significantly decreased growth in comparison to control, underscoring a biphasic role for NURR1 in breast cancer progression.

**Conclusions:**

NURR1 function presents a dichotomy in breast cancer etiology, in which NURR1 expression is associated with normal breast epithelial differentiation and efficacy of systemic cancer therapy, but silencing of which attenuates tumor growth. This provides a strong rationale for the potential implementation of NURR1 as a pharmacologic target and biomarker for therapeutic efficacy in breast cancer.

## Background

The NR4A family (NR4A1, NR4A2, and NR4A3) is a family of orphan nuclear receptors whose activity is shown to promote cell proliferation, apoptosis, and terminal differentiation in a tissue dependent manner [1]. All three family members have been shown to play roles in hematopoietic differentiation, while NURR1 (NR4A2, TINUR) activity is necessary for dopaminergic neuron differentiation [2-5]. Structural studies suggest that NR4As are “true orphan receptors”, in that the ligand binding pocket is thought to be obstructed by hydrophobic amino acid side chains rendering it inaccessible to ligands [6]. Despite the lack of a physiological ligand, NR4A receptors are targeted by several hormones and xenobiotic compounds which induce NR4A gene expression and/or directly bind to and elicit NR4A transactivation function [7-11]. Functionally, NR4As mediate gene expression by binding as monomers to NBRE [(NGFI-β Nerve growth factor inducible β) Response Element], as homodimers to NURRE (NUR-like Response Element), or as heterodimers with retinoid X receptor to DR5 response elements [12-15]. In addition to transactivation functions, NR4As have been shown to translocate to the mitochondria to induce apoptosis (NR4A1) and to modulate the activity of other proteins through protein-protein interactions (NURR1) [16-18].

Despite its role in differentiation, NURR1 has been implicated in promotion of cancer cell proliferation. Cytoplasmic localization of NURR1 is associated with decreased patient survival in bladder cancer patients while expression of NURR1 allowed HeLa retrovertant cell lines to regain tumorigenicity [19,20]. Additionally, prostaglandin-mediated cytoprotection has also been shown to be dependent on NURR1 expression [21]. Similarly, thromboxane A mediated lung cancer cell proliferation is in part mediated through NURR1 [22]. Conversely, drugs which transactivate NURR1 have been shown to be associated with apoptosis. For instance, NURR1 has been identified as a target of the anti-neoplastic drug 6-mercaptopurine, and may contribute to its anti-neoplastic functions, while 1, 1-bis(3′-indolyl)-1-(p-chlorophenyl)methane (DIM-C-pPhCl), an activator of NURR1 has been shown to mediate apoptosis in bladder cancer cells [10,23].

Despite these findings, the impact of NURR1 expression has yet to be elucidated in breast cancer. In order to gain insight into the function of NURR1 in breast cancer, we performed immunohistochemical staining for NURR1 on breast tissue arrays and compared the expression of NURR1 protein in normal vs. transformed breast tumor samples. Furthermore, we compared the level of NURR1 expression among tumor samples stratified according to lymph node status, histological grade, estrogen receptor α (ERα) status, and p53 expression status. To support these findings, we codified NURR1 expression in several publically available microarray datasets in which normal breast epithelium was compared to cancerous breast. Relapse free survival (RFS) of patients exhibiting high or low NURR1 expression was compared to determine the association of NURR1 with breast cancer recurrence. Additionally, we developed a xenograft model to determine the impact of NURR1 silencing in breast tumor development. These studies support the contention that NURR1 could be an efficacious target in cancer chemoprevention and therapy, as well as a potential biomarker for treatment efficacy in breast cancer.

## Methods

### Breast tissue microarrays and immunohistochemistry

Breast tumor microarrays (BR953) were purchased from US Biomax incorporated for immunohistochemical staining. Briefly, slides were deparaffininzed at 60°C and incubated in xylenes for 3 minutes. Slides were subsequently rehydrated by incubation in graded ethanol at 100%, 90%, and 75%. Heat-induced epitope retrieval (HIER) was performed in an autoclave at 100°C, for 10 minutes, at 15 PSI, in 20mM Tris, pH 8.5. Antibodies used for immunohistochemical staining included normal rabbit IgG (negative control) or α-NURR1 (N-20, Santa Cruz Biotechnology). Peroxide block, blocking, antibody incubation, and secondary detection were performed utilizing UltraVision One Polymer IHC detection systems (Thermo Scientific) in accordance with the manufacturer’s instruction. Stained core images were captured using an Olympus BX51 and DP72 color camera. Cores were each scored according to staining intensity (0 = negative, 1 = marginal/weak, 2 = moderate, 3 = strong) twice each by 2 blind observers and the mean scores recorded. Mean IHC scores of normal and cancerous epithelium were compared using Mann-Whitney U-test. For further analyses, biopsies with a mean score of less than 1.5 were scored as NURR1(-), and those at 1.5 or above were designated as NURR1(+). Utilizing pathology reports, patient data was stratified according to TP53 expression, ERα expression, lymph node status, and histological grade. Statistical significance was determined using Fisher’s exact test. Images of histological (hematoxylin and eosin) stains for each tumor core are available at http://www.biomax.us/tissue-arrays/Breast/BR953.

### Western immunoblot

For Western immunoblots, normal and cancerous tumor lysates were purchased from Origene technologies. Lysates from established cell lines (MDA-MB-468 and MDA-MB-231) cell lines were generated from 60% confluent 100 cm^2^ cell culture plates using 1% SDS buffer supplemented with protease and phosphatase inhibitor cocktail. All protein lysates were fractionated by polyacrylamide gel electrophoresis (PAGE) and transferred to nitrocellulose. Nitrocellulose blots were blocked and probed in the presence of 5% bovine serum albumin for the presence of α-NURR1 and a β-actin antibodies. Secondary immunodetection was performed by incubation with AlexaFluor-647 or AlexaFluor-488 secondary antibodies, respectively. Immunofluorescence was detected using the BioRad VersaDoc imaging system.

### Tissue culture and cell line generation

MDA-MB-468 and MDA-MB-231 cells were acquired from American Tissue Type Collection (ATCC) and utilized to generate novel cell lines (4A2KD-468, 4A2KD-231, Vec-468, and Vec-231). 4A2KD- and Vec- cells lines were generated by stable transfection with plasmids (pGFP-V-RS vector, Origene) expressing scrambled short hairpin RNA (shRNA) or a shRNA targeting NURR1 (Vec- and 4A2KD- cells, respectively) using Fugene HD (Roche) in accordance with the manufacturer’s protocols. Stably transfected cells were selected by FACS (fluorescence assisted cell sorting) gating according to GFP fluorescence at 5 days post-transfection (Tulane University Cell Analysis Core). All cell lines were cultured in Dulbecco’s modified eagle’s media (DMEM), supplemented with 10% fetal bovine serum and penicillin/streptomycin, and maintained at 37°C and 5% CO_2._

### Tumor xenografts

Four- to five-week old female homozygous athymic nude mice (Hsd-nude-Foxn1^nu^, approximately 20 grams each) were purchased from Harlan Laboratories. After 10 days quarantine, each mouse was identified by numbered ear tags and randomly assigned to 4 cage groups with 6 mice each: Vec-468, 4A2KD-468, Vec-231, and 4A2KD-231. The GFP-expressing cells were cultured to 80% confluence in 150 mm^2^ tissue culture plates, then collected and divided into aliquots containing 5×10^5^ cells with Matrigel suspension. Each mouse was inoculated once by injection of cell/Matrigel suspension (200 μl) into the inguinal mammary fat pad with 5×10^5^ cells. After day 10, tumors were imaged for GFP fluorescence using the Maestro Flex small animal imager (day 0), and then weekly for five weeks thereafter to measure tumor progression as indicated by fluorescence intensity. During imaging, mice were anesthetized (intraperitoneal injection with ketamine/xylazine) to immobilize the animals during image acquisition. Images were spectrally unmixed and fluorescence totals reported. Upon termination of the study, mice were euthanized by exposure to CO_2_ in a manner as to minimize animal distress. All animals were housed in the Animal Care Facility on-site and received humane care according to the guidelines of the Institutional Animal Care and Use Committee (IACUC) of Xavier University of Louisiana.

### Data mining

GEO microarray array public repository was initially searched for microarrays in which global gene expression in normal breast epithelium was compared to that of cancerous breast tissue. Within the search, 5 studies were identified. NURR1 expression values were derived from each dataset, and relative expression of NURR1 was compared using Student’s T-test for significance, where significance was determined as p ≤ 0.05.

Kaplan-Meier survival analysis was performed utilizing kmplotter server (kmplot.com), which analyzes breast cancer patient survival data from public microarray data repositories. Patients were stratified as NURR1-low or NURR1-high according to the median expression values for NURR1 throughout the cohort (Affymatrix probe 216248_s). RFS in the total population (2898 patients) was determined and compared to that of patients which did not receive systemic therapy (845 patients).

## Results

### NURR1 is strongly expressed in normal, but not cancerous breast tissue

**Figure 1:**
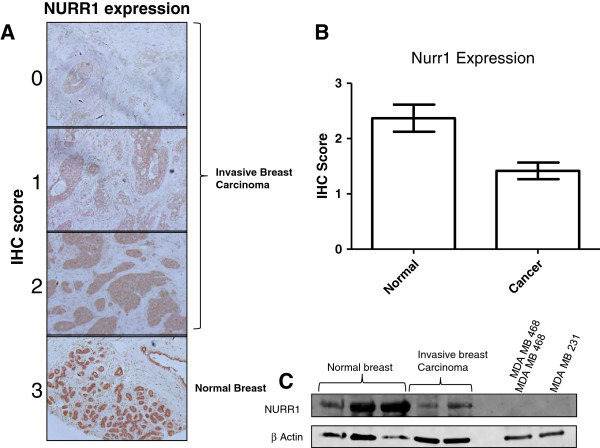
**NURR1 is weakly expressed in cancerous breast epithelium, but strongly expressed in the normal breast epithelium. A**) Immunohistochemical staining of breast tissue microarrays reveals expression of NURR1 in tissue cores correlating to normal and malignant breast. Images typical of scoring are depicted, with normal breast tissue shown in the lower panel.** B**) Each tissue core was scored independently and the mean for normal and cancerous epithelium compared using Mann-Whitney U-Test, p = 0.0016. Hematoxylin and eosin staining of each core is available at http://www.biomax.us/tissue-arrays/Breast/BR953**C**) NURR1 expression was determined by Western immunoblot of protein lysates derived from normal breast epithelium, cancerous breast epithelium, and established breast cancer cell lines (MDA-MB-468, MDA-MB-231).

NURR1 impacts proliferation and differentiation in a context-dependent fashion. As such, we sought to compare the expression of NURR1 in normal and cancerous breast epithelium using tissue microarrays. Tissue microarrays were stained with antibodies directed against NURR1 or with normal IgG, and each tissue core was scored according to the intensity of NURR1 staining in epithelial cells (0 = absent and 3 = greatest intensity). NURR1 was strongly expressed in normal breast epithelium, with a mean intensity score of 2.4 (Figure 1A, B). This differed significantly from cancerous tissue cores which had a mean intensity score of 1.4 (Figure 1A, B). This specific silencing of NURR1 in transformed breast was confirmed in using Western immunoblots, where relative NURR1 expression was determined in lysates from normal breast epithelium, transformed breast, and established cell lines (MDA-MB-468 and MDA-MB-231). Loss of NURR1 expression is evident in cancerous breast as compared to normal breast, (Figure 1C). These findings support the contention that NURR1 expression in the breast is commensurate with a normal, terminally differentiated epithelial phenotype, whereas as silencing/dysregulation of NURR1 may play a role in oncogenic transformation of breast epithelial cells.

### NURR1 expression is associated with specific prognostic indices in breast cancer

**Figure 2:**
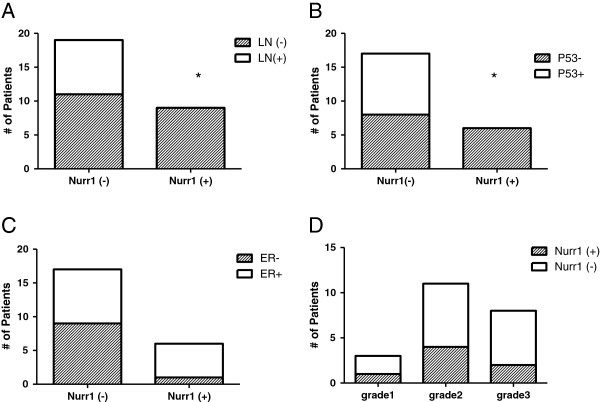
**NURR1 protein expression correlates with specific prognostic indicators in breast cancer biopsies.** Breast tumor microarray cores which were previously scored were categorized as either NURR1(-) (mean IHC score, < 1.5) or NURR1(+) (mean IHC score, ≥1.5). Subsequently, each category was compared with regard to several clinicopathological features (lymph node status, p53 status, ERα expression) using Fisher’s Exact Test. NURR1 is silenced in the primary tumors of patients with lymph node metastasis (**A**, p = 0.0292) and is inversely correlated to p53 expression (**B**, p = 0.0481). NURR1 status did not significantly correlate with ERα status (**C**, p = 0.1790) or histological grade (**D**, p = 0.928).

Since NURR1 showed a highly significant decrease in expression in breast cancer versus normal breast, we investigated whether NURR1 was associated with specific surrogate prognostic indices among breast cancer patients. Breast tumors were categorized into groups of either negative to marginally expressing NURR1 [NURR(-)] and those moderately to strongly expressing NURR1 [NURR(+)], based on previously determined mean IHC scores (0.0-1.49, 1.50-3.0, respectively). Subsequently, each sample was categorized according to accompanying pathology reports with regard to ERα status, p53 status, presence of lymph node metastases (from TNM classification), and histological grade. NURR1 expression was strikingly lower among primary tumors of patients with lymph node metastases (Figure 2A). Indeed each of the primary tumors associated with lymph nodes was NURR1(-) suggesting a strong link between NURR1 silencing and cancer cell invasiveness (Figure 2A). Additionally, we found that NURR1 expression was inversely related to expression of p53 (Figure 2B). Though the correlation was not of statistical significance, NURR1(-) tumors tended to be ERα(+) (Figure 2C). No differences were observed between the histological grades represented with regard to NURR1 expression (Figure 2D).

### Data mining confirms NURR1 silencing in transformed breast as compared to normal breast epithelia

To confirm that NURR1 expression is selectively silenced in transformed breast cells, we analyzed published gene expression microarrays in the Gene Expression Omnibus (GEO) repository which entailed comparisons of normal and transformed breast epithelium. We then compared the expression of NURR1 between cancerous and normal patient samples. Four of five studies revealed decreases in mean NURR1 mRNA expression in transformed breast cancer samples as compared to normal breast epithelium, three of which reached statistical significance, p ≤0.05 (Table 1) [24-28]. This supports our own breast tissue microarrays findings demonstrating that loss of NURR1 expression is indeed associated with oncogenic transformation of the breast epithelium. Interestingly, those cohorts showing the most significant difference appeared in studies in which normal tissue was derived from breast reduction mammoplasty where no diagnosis of breast cancer had been made. Conversely, the 2 datasets (GDS2739 and GDS2635) which showed no statistically significant difference in NURR1 expression, involved use of tumor-adjacent normal breast tissue. As such, it is plausible that the loss of NURR1 expression is not only a marker for breast epithelial cell transformation, but may be a marker of pre-neoplastic breast epithelium.

**Table 1:** NURR1 expression in gene expression microarrays comparing normal and cancerous breast epithelium

**GEO dataset**	**Normal**	**Cancer/Hyperplasia**	**T-Test**	**PMID**
GDS3139	1942 _± 396.6 N=15_	417.8 _± 35.81 N=14_	P = 0.0010	18058819
GDS2739	1691 _± 943.0 N=8_	487.2 _± 115.3 N=8_	P = 0.2257	17591970
GDS3716	1718 _± 265.2 N=18_	535.6 _± 108.7 N=18_	P = 0.0002	20197764
GDS2635	327.3 _± 93.02 N=19_	296.2 _± 59.50 N=11_	P = 0.8143	17389037
GDS2250	7.780 _± 0.3715 N=7_	6.290 _± 0.2538 N=40_	P = 0.0223	16473279,20400965

### NURR1 is associated with prolonged RFS in breast cancer patients

**Figure 3:**
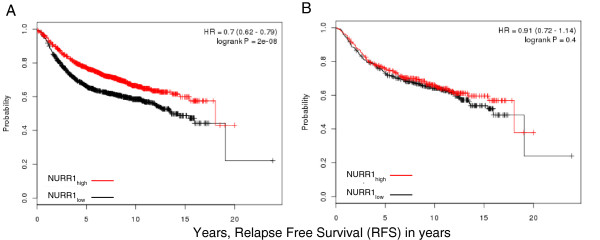
**NURR1 expression is associated with increased relapse free survival (RFS).** Survival of patients expressing above median NURR1 mRNA (NURR1_high_) was compared to patients with below median expression (NURR1_low_) levels of NURR1 mRNA. **A**) Among all patients, those categorized as NURR_high_ had a substantial RFS benefit as compared to NURR1_low_ (HR = 0.7, N = 2989) **B**) The RFS benefit observed among NURR1_high_ patients was not apparent in patients which did not receive systemic therapy for the treatment of breast cancer (HR = .91, N = 845).

Lymph node metastases and p53 expression serve as surrogate markers for breast cancer prognosis, but may not correlate directly with patient survival. As such, we utilized the kmplot.com server to determine the relationship between NURR1 expression and RFS in breast cancer patients [29]. Using the JETset best probe function, we identified Affymatrix probe 216248_s_at as the most suitable probe for determination of NURR1 expression. Patients were designated as NURR-high or NURR1-low based on the median expression of NURR1 within the complete cohort. Kaplan-Meier analysis of 2898 breast cancer patients reveal that NURR1 is significantly associated with improved prognosis as determined by RFS, where HR = 0.7 (0.62-0.79, LogrankP = 2×10^-8^) (Figure 3A). However, when limited to patients not receiving systemic therapy (846 patients), NURR1 failed to reveal any RFS advantage [HR = 0.91 (0.72-1.14, logrankP =0.40) (Figure 3B). This suggests that the positive prognostic significance of NURR1 may be less related to disease progression, but more indicative of the efficacy of systemic therapy in prolonging time to relapse.

### NURR1 silencing inhibits breast tumor xenograft growth

**Figure 4:**
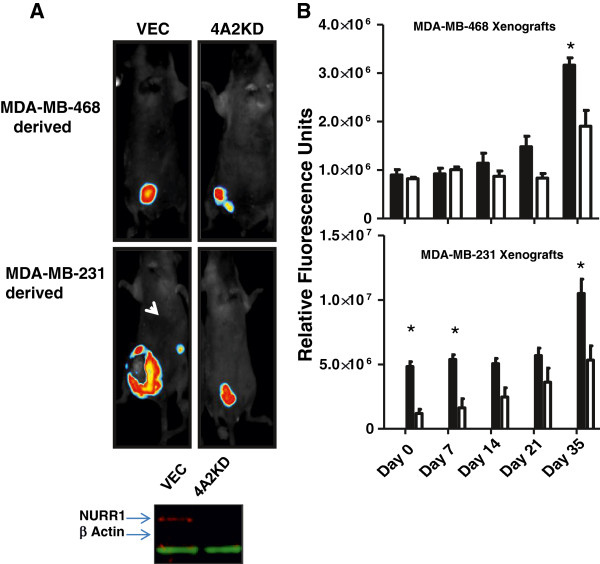
**Silencing of NURR1 expression inhibits in growth of mammary xenograft tumors. A**) Cell lines stably transfected with plasmids expressing GFP with (4A2KD-) or without (Vec-) shRNA against NURR1 were generated from MDA-MB-468 and highly invasive MDA-MB-231 breast cancer cell lines. NURR1 silencing efficiency was ascertained by Western blot. Athymic nude mice were inoculated by injection with 5×10^5^, and tumor growth was monitored over 35 days by GFP fluorescence using a Maestro Flex small animal imager (Vec-468 and 4A2KD-468 upper panels, Vec-231 and 4A2KD-231 lower panels). In MDA-MB-231 derived cell lines, stable silencing of NURR1 resulted in decreased contralateral metastases (arrow). **B**) Quantification of the tumor associated fluorescence was quantified, and NURR1 silencing resulted in significant (*two-way analysis of variance, p < 0.01) inhibition in tumor growth by 35 days post inoculation in both cell lines).

In order to determine the impact of NURR1 silencing on breast cancer cell proliferation, we stably transfected MDA-MB-468 and MDA-MB-231 cells with a short hairpin RNA (shRNA) encoding plasmids directed toward either NURR1 or a scrambled hairpin RNA as a control, (4A2KD- and Vec-, respectively), resulting in suppression of NURR1 protein expression in 4A2KD-468 and 4A2KD-231 cells. In addition, each plasmid also encoded green fluorescent protein (GFP), allowing efficient determination of stable transgene incorporation and monitoring of tumor growth. We subsequently established orthotopic mouse tumors in Hsd-Nude-foxn1^nu^ athymic nude mice; after which the tumors were allowed to progress for 35 days and the tumor size was monitored by GFP fluorescence. A divergence in tumor growth rate was observed by day 14 and by day 35, Vec-468 tumors had grown at a significantly faster rate and were 60% larger than 4A2KD468-tumors, Figure 4A, B). 4A2KD-231 cells were more efficient in establishing tumors, and by day 35 showed a substantial increase in growth as compared to Vec-231 derived tumors. Additionally, 4 of 6 mice inoculated with Vec-231 cells developed detectable contralateral mammary lesions whereas none were detected among mice inoculated with 4A2KD-231 cells, highlighting a potential role for NURR1 expression in breast cancer cell invasion. When taken into consideration with previous findings, these data suggest a dichotomous role for NURR1 in breast cancer development in which NURR1 is highly expressed in normal, non-proliferating breast epithelium, but acquires proliferation promoting effects in transformed tissue.

## Discussion

Our studies suggest that NURR1 has profound, context dependent effects on breast cancer and normal breast epithelium with regard to tumorigenicity and terminal differentiation, respectively. We have demonstrated that NURR1 is strongly expressed in the normal breast epithelium, but is suppressed in the transformed breast, suggesting a potential role for NURR1 in the maintenance of a differentiated epithelial phenotype. Our studies also reveal that NURR1(-) primary tumors are more likely to be associated with p53 expression as well as increased incidence of lymph node metastases, when compared to NURR1(+) tumors. Paradoxically, breast cancer xenograft tumors in which NURR1 has been targeted by stable transfection with shRNA reveal that further loss of NURR1 leads to decreased tumor growth as compared to control. These findings suggest a dichotomous role for NURR1 in breast cancer development which may be substantially impacted by the cellular context under which the receptor is expressed.

### NURR1 in cancer

The contention that NURR1 plays an important role in the maintenance of terminal differentiation of epithelia is supported in the literature. Developmental animal models have shown that suppression of NURR1 is necessary for the maintenance of pluripotency of hematopoietic progenitor cells [3]. Similarly, genetic models have demonstrated that the loss of NR4A-family receptors results in increased incidence of leukemia [30]. NURR1 expression and activity is induced in response to several compounds with anti-neoplastic effects such as 6 mercaptopurine and 1,1-bis(3′-indolyl)-1-(aromatic)methane (C-DIM) analogs [7,10]. In contrast, several studies suggest that NURR1 is associated with increased proliferation of cancer cells. In HeLa cells, loss of NURR1 was associated with decreased anchorage independent growth and resulted in apoptosis, suggesting that NURR1 was necessary for the maintenance of a tumorigenic phenotype [20]. In colon cancer, it has been demonstrated that prostaglandin E2 mediated proliferation is inhibited by expression of a dominant negative NURR1, demonstrating that NURR1 is indeed necessary for eicosanoid-mediated proliferation in colon cancer [21]. It is feasible that NURR1, which is highly regulated at the transcriptional and post-translational levels, may have different roles in cancer based on the regulatory influences present within the cellular environment. To this point, NURR1 mislocalization to the cytoplasm is associated with poor clinical prognosis, yet pharmacological modulation of the transcriptional function of NURR1 is associated with compounds which induce apoptosis [10,19]. Together, these findings suggest that nuclear localization, and presumably transcriptional activity of NURR1 is associated with differentiation, whereas a cytosolic localization and a lack of transcriptional activation are supportive of tumorigenesis. Indeed, our own immunohistochemical studies suggest that in normal cells, NURR1 is strongly localized to the nuclear compartment, supporting the contention that NURR1 has differential roles in the normal and transformed breast. Therefore, expression of the receptor alone may not be indicative of its role in cancer, but its transcriptional activity may be the key to elucidating its role in the inhibition or promotion of breast cancer. Further elucidation of this potential mechanism is complicated by the fact that breast cancer cell lines are often intolerant of NURR1 overexpression (data not shown). Therefore, functional studies involving the transfection of wild-type and transcriptionally inactive variants of NURR1 will likely require a more nuanced approach, such as conditional overexpression models.

### NURR1 and breast cancer

In our studies, we have found that NURR1 silencing is associated with increased incidence of lymph node metastasis, and is associated with decreased expression of the tumor suppressor p53. Strikingly, no NURR1(+) tumors were associated with lymph node metastases, which supports the notion that NURR1 functions as a tumor suppressor. The prognostic significance of the inverse relationship between NURR1 and p53 expression is unclear, but may yield some insight into the potential mechanistic role of NURR1 in prevention of cancer development. One intriguing possibility is that NURR1 serves as a regulatory point secondary to p53 thus preventing entry into the cell cycle in the absence of p53 expression. A complicating factor however, is that p53 is frequently mutated in cancer, thereby making it difficult to assert whether p53 is active when it is expressed in breast cancer [18].

Based on the findings in tissue arrays, it might be expected that experimental silencing of NURR1 would result in an increase in tumor development and growth. To the contrary, our observation that NURR1 silencing caused a decrease in tumor growth in two xenograft models suggests that NURR1 acquires a tumor promoting function when expressed in transformed cells. It therefore is unlikely that the tumor promoting effect of NURR1 is a passive effect of inactive NURR1. Instead, we contend that NURR1 actively contributes to oncogenic signaling under currently undefined cellular conditions that could include posttranslational modification of NURR1, protein-protein interactions, or differential transactivation function of NURR1. Interestingly, mRNAs encoding splice variants of NURR1 have been characterized, and several of these presumed gene products have dominant negative effects with regard to NURR1 –dependent transcriptional activity [31,32]. As mentioned above, our early attempts to transiently overexpress NURR1 in breast cancer suggest that breast cancer cells are intolerant to NUR1 expression, resulted in rapid cell death (data not shown). If taken into context with these findings, this suggests that there may be some threshold or “gene-dose” effect of NURR1 on proliferation/survival in cancer, where low NURR1 expression levels may support proliferation, but higher levels of expression may lead to cell cycle arrest or cell death through distinct mechanisms

## Conclusions

From these studies, we conclude that NURR1 expression and transactivation is an integral component of normal breast epithelial differentiation and functions as an indicator for the efficacy of systemic therapy in breast cancer. Additionally, we conclude that NURR1 acquires tumor promoting effects within the context of the cancerous breast, in which tolerable, low, significant levels of NURR1 expression support breast tumor development.

## Abbreviations

NURR1: Nuclear receptor related 1; TINUR: Transcriptionally inducible nuclear receptor; ERα: Estrogen receptor α; RFS: Relapse free survival; HR: Hazard ratio; NBRE: NGFIβ response element; NuRE: Nur-like response element; IHC: Immunohistochemistry; GFP: Green fluorescent protein; IgG: Immunoglobulin G

## Competing interests

The authors declare that they have no competing interests.

## Authors’ contribution

SL and LC established cell lines, performed tissue culture as well as established and quantified mouse xenograft studies. TD and BS scored immunohistochemical staining of tissue arrays for NURR1 expression. BR contributed to the conceptualization of the project as well as material support. CW directed all activities, as well as performed immunohistochemical staining, and data mining of public microarray repositories for expression and RFS data. All authors read and approved the final manuscript.

## Pre-publication history

The pre-publication history for this paper can be accessed here:

http://www.biomedcentral.com/1471-2407/13/139/prepub
